# The Impact of CYP3A5 Genotype on Tacrolimus Pharmacokinetics in Children following Heart Transplant

**DOI:** 10.21203/rs.3.rs-8780228/v1

**Published:** 2026-02-12

**Authors:** Joseph Rower, Tia Freeman, Cassandra Deering-Rice, Kimberly Molina

**Affiliations:** University of Utah

**Keywords:** Pediatrics, Heart Transplant, Tacrolimus, Pharmacogenetics, CYP3A5

## Abstract

Tacrolimus is a first-line immunosuppressant used after solid organ transplantation that suffers from extensive intra- and inter-patient variability and a narrow therapeutic window. Tacrolimus clearance is predominantly mediated by CYP3A4 and CYP3A5, with evidence indicating that pharmacogenetic (PG) differences in these enzymes drive significant changes in tacrolimus pharmacokinetics (PK) and therapeutic tacrolimus dosing. The current study investigates the hypothesis that CYP3A4/5 genetic variation alters tacrolimus dose requirement and metabolite concentration in children following pediatric heart transplant. Saliva and whole blood samples were collected from children who previously received a heart transplant as part of a tacrolimus PG/PK study. A total of 64 children were included in the analysis, which found that children carrying a CYP3A5*1 allele required a 2.7-fold higher tacrolimus dose (p<0.0001) than individuals with the CYP3A5*3/*3 genotype. Dose-normalized tacrolimus and metabolite concentrations were significantly lower in children with the CYP3A5*1 allele. Combined, these data support the impact of CYP3A5 genotype on tacrolimus PK and demonstrate the need for larger studies to confirm the clinical importance of this genotype for appropriate tacrolimus dosing in pediatric heart transplant recipients.

## Introduction

Heart transplantation is an accepted therapeutic option for children with congenital heart disease and cardiomyopathy.^1^ Nearly 500 pediatric heart transplants were performed in 2022 across the United States with improving outcomes in recent decades, though mortality from rejection, infection, and coronary vasculopathy remain significant.^2,3^ Transplant survival in excess of 20 years following heart transplantation has been observed, with more than 70% of transplants expected to achieve greater than 5 year survival.^2,4^ Much of this success can be attributed to the use of immunosuppressive therapy to prevent the rejection of the transplanted cardiac tissue. Tacrolimus is used as a primary immunosuppressive option but suffers from extensive inter- and intra-patient pharmacokinetic (PK) variability.^5^

Tacrolimus is cleared via liver metabolism, primarily mediated by cytochrome P450 (CYP) 3A5, along with CYP3A4.^6,7^ Polymorphisms in the genes encoding these two drug metabolizing enzymes may contribute to the inter-patient PK variability observed for tacrolimus. For example, the CYP3A5*3 allele (rs776746) common in the Caucasian population (allele frequency >80%) yields non-functional protein, with homozygous CYP3A5*3/*3 individuals requiring lower tacrolimus doses to achieve therapeutic concentrations than those with a CYP3A5*1 allele.^8,9^ Similarly, CYP3A4*22 (rs35599367) has previously been associated with reduced CYP3A4 mRNA expression and enzyme activity, along with a resulting decrease in tacrolimus dose requirement in individuals carrying this allele.^10–13^ Despite numerous reports of the impact of these genotypes on tacrolimus PK, pharmacogenetic guided tacrolimus dosing is not widely implemented in the clinical setting.^5,6,9,10,12,14–25^

The purpose of this study was to understand the impact of the *CYP3A5* and *CYP3A4* genotypes on tacrolimus PK and dose requirement following pediatric heart transplant, to further enable individually optimized tacrolimus dosing in this population. Few papers have evaluated the impact of genetics within the pediatric heart transplant population,^19,20,22^ and none have associated genotype with concentrations of tacrolimus metabolites in this population. Additionally, we sought to incorporate the impact of these genotypes into our previously described population PK model, in an effort to improve the accuracy and efficiency of our published decision support tool^26^ for guiding tacrolimus dosing in this population.

## Methods

### Study Population

Study approval was granted by the University of Utah, Intermountain Health, and Primary Children’s Hospital Institutional Review Board (IRB_00124800, NCT04380311). All study procedures followed the ethical guidelines outlined in the Declaration of Helsinki. Children and their parent(s) were approached by study staff to discuss participation in a clinical study focused on the impact of genetics on tacrolimus PK at a standard-of-care outpatient clinic visit between 2020 and 2023. Parental permission was obtained for all participants, and assent was obtained from participants greater than 7 years of age. Eligibility criteria included being between the ages of 6 months and 17 years of age, the receipt of a heart transplant due to diagnosis of a congenital heart malformation or cardiomyopathy, and the use of a tacrolimus based immunosuppressive treatment regimen. The study targeted enrollment of at least 60 children, with *a priori* power calculations indicating a need to identify 20 children with the CYP3A5*1/*x genotype to have ~90% power to distinguish a significant difference (>1.8-fold change) in dose-normalized tacrolimus concentrations.

### Sample Collection

After completion of the consent process, a single buccal swab sample was collected for genotype analyses using a DNA/RNA Shield SafeCollect Saliva Collection Kit (Zymo Research, Irvine, CA, USA) prefilled with a DNA stabilizing agent. The child was asked to chew on their cheeks and tongue to increase sample yield prior to collection. A cotton swab was then rubbed inside the child’s cheek, gums, and on their tongue to collect buccal cells for genomic DNA isolation. The swab was then stored in preservation media within a sealed tube at −30°C until analysis.

One additional 2mL blood sample was collected from study participants during the venipuncture sampling used to collect standard-of-care safety laboratory and tacrolimus therapeutic drug monitoring (TDM) samples at each of four clinic visits. These samples represent morning trough concentrations. The extra sample was processed by the clinical laboratory to generate both a whole blood sample (~0.5 mL) and plasma samples (5 × ~0.1 mL aliquots), which were then stored at −80°C until analysis. The current study focuses on data collected from the whole blood sample, while the plasma samples were reserved for metabolomic and lipidomic analyses.

### Genotyping

The collection of genomic DNA (gDNA) and genotyping analyses have been described previously.^27^ Briefly, genomic DNA was isolated from 2 mL of the samples using the PureLink gDNA mini kit (ThermoFisher, Carlsbad, CA, USA) per the manufacturer’s protocol. *CYP3A5* allele status was determined from 15 ng of gDNA using the TaqMan SNP genotyping enzyme (ThermoFisher, Carlsbad, CA, USA), probing for rs776746 (assay ID: C_26201809_30). CYP3A4*22 (rs35599367) allele status was also determined using assay ID: C_59013445_10. TaqMan reactions were cycled as recommended by the manufacturer on an Applied Biosystems QuantStudio6 instrument. Data clustering analysis was performed using TaqMan Genotyper software v1.7.1 (Applied Biosystems by ThermoFisher, Carlsbad, CA, USA).

### Bioanalysis

Tacrolimus concentrations for clinical TDM were determined from whole blood using a validated liquid chromatography-tandem mass spectrometry (LC-MS/MS) method at ARUP^®^ Laboratories. The assay was linear between 1 and 40 ng/mL.

Tacrolimus and metabolite concentrations were determined using the additional whole blood sample collected specifically for research study procedures. Tacrolimus was purchased from Cerilliant (Round Rock, TX, USA), Tacrolimus-^13^CD_2_ was purchased from Cayman Chemical (Ann Arbor, MI, USA) for use as internal standard, while the 13-O-desmethyl (M-I) and 31-O-desmethyl (M-II) metabolites were kindly provided by iC42 Clinical Research and Development (Aurora, CO, USA). After addition of the internal standard (tacrolimus-^13^CD_2_ was used for all analytes), a 100 μL aliquot of whole blood was extracted using a liquid-liquid extraction approach. Briefly, 20 mM zinc sulfate was used to lyse red blood cells, followed by addition of 2mM ammonium acetate (pH 5.3) to modify pH and 2 mL of methyl *tert*-butyl ether (MtBE) as the extraction solvent. The sample was then vortexed, centrifuged, and frozen at −80°C, before decanting the MtBE into a fresh 13 × 100 polypropylene tube. The MtBE was dried in a Zymark TurboVap under house air (~15 psi, 40C, 15 min), reconstituted in 200 μL 50:50 2mM ammonium acetate (pH 5.3, aq):2mM ammonium acetate in methanol with 0.1% formic acid, and transferred to a 300 μL polypropylene autosampler vial for LC-MS/MS analysis.

The LC-MS/MS consisted of a ThermoScientific TSQ Quantis Plus (San Jose, CA, USA) triple quadrupole mass spectrometer coupled with a ThermoScientific Vanquish Flex ultra-high performance LC (UHPLC) pump and autosampler. Chromatographic separation was achieved with a Phenomenex (Torrance, CA, USA) Gemini C18 column (150 × 2 mm, 5μm) held at 45°C and a gradient mobile phase consisting of (A) 2mM ammonium acetate (pH 5.3, aq) and (B) 2mM ammonium acetate in methanol with 0.1% formic acid flowing at 0.25 mL/min. Initial mobile phase conditions were held at 75% B for the first minute of the injection, then linearly increased to 85% B for the next 1.5 minutes, followed by a linear increase to 100% B for the next 1. 5 minutes, which was held for another 2.5 minutes before returning to and reequilibrating at initial conditions. Monitored mass transitions (collision energy) in positive electrospray ionization mode were 821.7 -> 768.4 (163 V) for tacrolimus, 824.6 -> 771.3 (164 V) for tacrolimus-^13^CD_2_, 812.6 -> 602.2 (299 V) for M-I, and 807.9 -> 754.4 (159 V) for M-II. A 1/x^2^ weighted linear regression was used to fit a calibration curve between 1 and 50 ng/mL for all analytes. Inter-assay accuracy (as % difference from nominal concentration, %diff) was within ±8.5% and precision (as % coefficient of variation, %CV) within 5.1% for tacrolimus across three batches of six replicate quality control samples prepared at three different concentrations. QC performance was more variable for the metabolites, however, the %diff was within ±13.6% and ±18.9% for the M-I and M-II metabolite, respectively, passing the *a priori* research method qualification acceptance criteria of ±20% set for the metabolites.

### Statistical and Pharmacokinetic Analyses

Basic statistical analyses utilized GraphPad Prism v10.6.1. Comparison of dose and concentrations with genotype used unpaired *t*-tests, with a significance level set at α=0.05. Concentration-genotype relationships were analyzed both with and without dose normalization; dose normalization was based on the weight-normalized total daily tacrolimus dose. The association of clinical TDM tacrolimus concentrations with tacrolimus concentrations quantified in-house utilized linear regression. A receiver operating characteristic (ROC) curve was utilized to determine a tacrolimus:M-I ratio that predicts genotype with sensitivity and specificity >80%.

We previously described the construction of a population PK model, which identified the covariates age, fluconazole use, and creatinine clearance as significant covariates impacting tacrolimus PK in this study population.^28^ However, *CYP3A4/5* genotypes were not available during this initial analysis, and were therefore tested within the model as part of the current study. Briefly, PK modeling utilized NONMEM software (v7.3, ICON Development Software, Ellicott City, MD, USA) interfaced with PDx-Pop (v5.0). The first order conditional estimation with interaction (FOCE-I) method was used throughout model building and evaluation. Model selection was based on parsimony, objective function value (OFV), and visual diagnostic plots. The model was parameterized on elimination rate (k_e_), volume of distribution (V), and a fixed absorption rate (k_a_=3.43 hr^−1^). Residual error used an additive model and between subject variability was included on k_e_ and V. The significance of *CYP3A5* genetics within the model was assessed both univariately and with the previously described covariates included *a priori* using a χ^2^-test based on nested model OFV.

## Results

### Study Population

A total of 65 children were enrolled in the study, ranging in age from 0.77 to 17.8 years of age. One child discontinued tacrolimus at their consent visit, so was excluded from the remainder of the study and the presented analyses. Summary demographic data for the 64 participants included in the analyses are described in Table 1. Of the children in the study, 44 were male, 20 were female, 59 were White, and 3 were Black. Two individuals were found to be homozygous for the CYP3A5*1/*1 allele, while 14 children were heterozygous (CYP3A5*1/*3). Only one participant was homozygous for the CYP3A4*22/*22 genotype, while 3 were heterozygous (CYP3A4*1/*22). Due to the paucity of individuals with the variant CYP3A4*22 allele, the impact of this genotype was unable to be interrogated further within the study.

Tacrolimus was administered as an oral capsule for 39 children, as a liquid suspension for 23 individuals, and as an extended-release oral capsule formulation for 3 participants. The median weight normalized daily dose was 0.0789 mg/kg/day, while the median tacrolimus concentration determined from clinical therapeutic monitoring was 6.0 ng/mL. A total of 221 whole blood samples were collected for analysis of tacrolimus, M-I, and M-II concentrations. Tacrolimus concentrations determined from an in-house assay developed at the Center for Human Toxicology (CHT) agreed well with results obtained from paired samples (n=213) by ARUP Laboratories^®^ for therapeutic drug monitoring purposes. A linear regression between the concentration results for the two assays yielded a slope of 1.04 (p<0.0001), with 92.5% of samples demonstrating an agreement within ±30%.

### *CYP3A5* Genetics and Dose Requirements

**Figure 1:**
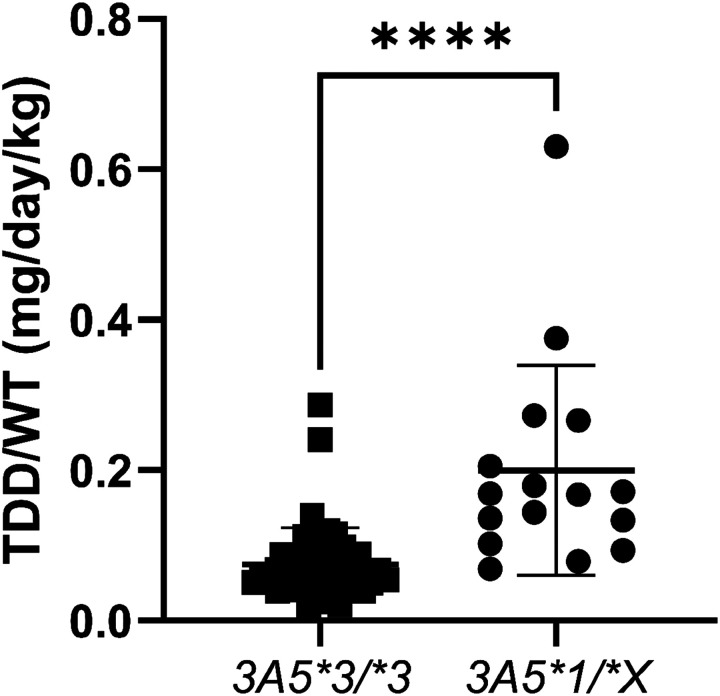
The weight-normalized total daily dose required to attain therapeutic tacrolimus concentrations are 2.7-fold greater in pediatric heart transplant recipients carrying a CYP3A5*1 allele compared to those with the CYP3A5*3/*3genotype. Asterisks denoting significance are defined as follows: * (0.05 ≥ p > 0.01), ** (0.01 ≥ p > 0.001), *** (0.001 ≥ p > 0.0001), and **** (0.0001 ≥ p). *Accessible Text:* A box and whiskers plot showing the weight-normalized total daily tacrolimus dose for pediatric heart transplant recipients with either the CYP3A5*3/*3genotype (left) or a genotype with the CYP3A5*1 allele (right).

The 16 children with at least one CYP3A5*1 allele received a 2.7-fold higher (p<0.0001) weight normalized total daily tacrolimus dose than individuals with the CYP3A5*3/*3 genotype (n=48, Figure 1). Two of the 3 highest weight-normalized total daily doses, regardless of genotype, were for children <3.5 months after transplant. Tacrolimus dosing guidelines vary by time after transplant, and recommend using doses that yield tacrolimus trough concentrations between 10 and 15 ng/mL for the first two months after transplant, between 8 and 12 ng/mL between 2 and 6 months after transplant, and between 5 and 10 ng/mL thereafter.^29^ The median (interquartile, IQR) time after transplant in our study population was 5.3 (1.9, 8.1) years, with 9 participants <6 months after transplant. Of these 9, 5 possessed the CYP3A5*1 allele. When these 9 participants were removed from the analysis (for receiving doses that target a different therapeutic range), the strong pharmacogenetic association remained (2.6-fold increase, p<0.0001).

### *CYP3A5* Genetics and Metabolite Concentrations

**Figure 2:**
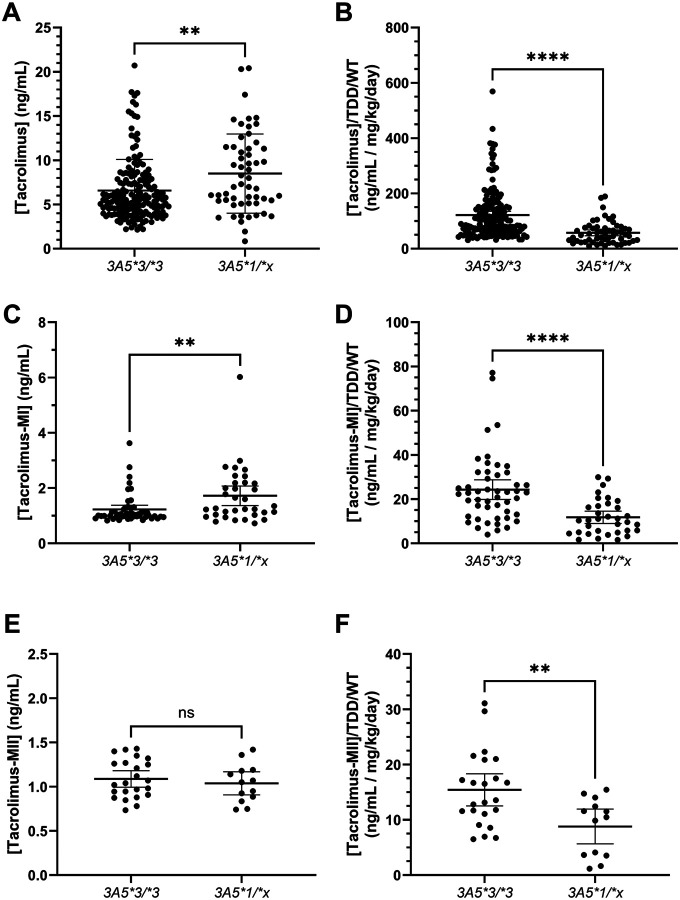
Concentrations of tacrolimus (a,b), tacrolimus M-I (c,d), and tacrolimus M-II (e,f) separated by *CYP3A5* genotype, with (a,c,e) and without (b,d,f) dose-normalization. Asterisks denoting significance are defined as follows: * (0.05 ≥ p > 0.01), ** (0.01 ≥ p > 0.001), *** (0.001 ≥ p > 0.0001), and **** (0.0001 ≥ p). *Accessible Text*: A box and whiskers plot showing concentrations of tacrolimus (a,b), tacrolimus M-I (c,d), and tacrolimus M-II (e,f) from left to right, separated by *CYP3A5*genotype (*3/*3 on the left, *1/*x on the right), and with (a,c,e) or without (b,d,f) dose normalization.

Mean (standard deviation, SD) tacrolimus concentrations were greater in children with the CYP3A5*1 allele compared to those with the CYP3A5*3/*3 genotype (8.49 (4.48) vs. 6.58 (3.50) ng/mL, p=0.0012, Figure 2a). When normalized by the weight-normalized total daily tacrolimus dose, this comparison flipped, with dose-adjusted tacrolimus concentrations higher in children with the CYP3A5*3/*3 genotype (p<0.0001, Figure 2b). Tacrolimus M-I concentrations were quantified in 83 of the 221 collected samples (37.6%), while tacrolimus M-II concentrations were quantified in only 36 of the 221 collected samples (16.3%). Tacrolimus M-I concentrations were quantifiable in 60.7% (n=34 of 56) samples from children with the CYP3A5*1 allele, but 29.5% (n=49 of 166) of samples from children with the CYP3A5*3/*3 genotype. Mean (SD) tacrolimus-M-I concentrations were 1.23 (0.541) ng/mL in children with the CYP3A5*3/*3 genotype and 1.72 (1.01) ng/mL in those with the CYP3A5*1 allele (p=0.0049, Figure 2c). When adjusted for weight-normalized total daily dose, the comparison again flipped, such that dose-adjusted tacrolimus M-I concentrations were higher in children with the CYP3A5*3/*3 genotype (p<0.0001, Figure 2d). Mean (SD) tacrolimus M-II concentrations were 1.09 (0.216) and 1.04 (0.216) ng/mL in children with the CYP3A5*3/*3 genotype and CYP3A5*1 allele, respectively (p=0.51, Figure 2e). Tacrolimus M-II concentrations were higher in children with the CYP3A5*3/*3 genotype relative to those with the CYP3A5*1 allele (p=0.0043, Figure 2f) when adjusted for weight normalized total daily dose. The ratio of tacrolimus to either tacrolimus M-I (p=0.057) and M-II (p=0.83) did not differ significantly by genotype.

Acknowledging that longitudinal data does not necessarily meet the independence criteria requiring for *t*-tests, we then subset the data to contain only baseline visits for each child and repeated the above analyses. In this analysis, many of the above comparisons lost significance. The comparisons that remained significant were dose-adjusted tacrolimus concentrations and unadjusted tacrolimus M-I concentrations, while the ratio of tacrolimus to its M-I metabolite attained significance, with the ratio ~33% lower in children with the CYP3A5*1 allele (p=0.02).

Finally, an ROC curve was utilized to determine that a tacrolimus:M-I ratio of 6.6 was associated with 87.5% sensitivity and 84.6% specificity to discriminate *CYP3A5* genotype. Specifically, tacrolimus:M-I ratios <6.6 are more likely to be associated with an individual carrying the CYP3A5*1 allele. The ROC curve had an area of 0.85 (p=0.0091), indicating the threshold’s predictivity is of moderately high strength.

### *CYP3A5* Genetics and Population PK Modeling

The impact of *CYP3A5* genetics was evaluated within a population PK model, combining the concentration data from the current study with previously published structural and error models.^28^ The univariate (i.e. without previously identified significant covariates) addition of *CYP3A5* genotype decreased OFV by 10.7 (p=0.0011), with parameter estimates indicating a 3.1-fold increase in the elimination rate of individuals with a CYP3A5*1 allele. Adding *CYP3A5* genetics to the full model (i.e. including previously identified significant covariates with the parameter estimates described in Rower et al.^28^) reduced the statistical significance of the association (ΔOFV = −6.1, p=0.014), but increased the magnitude of change associated with possessing the CYP3A5*1 allele to 5.4-fold. Diagnostic plots supported appropriateness of the model when *CYP3A5* genotype was included. A total of 17 children in the current study were included in the 45 participants previously studied.^28^ However, only 3 of these 17 possessed the CYP3A5*1 allele, preventing an interrogation of *CYP3A5* genotype within the dataset utilized to build the previously published population PK model.

## Discussion

This study demonstrates a clinically significant impact of *CYP3A5* genotype on tacrolimus PK, and thus, dose requirement, in pediatric heart transplant recipients. Children carrying at least one CYP3A5*1 allele required approximately 2.7-fold higher weight-normalized daily doses of tacrolimus to achieve therapeutic concentrations compared to those with the CYP3A5*3/*3 genotype. In addition to alterations in dose requirements, we observed genotype-related differences in tacrolimus metabolite concentrations that highlight the complexity of tacrolimus disposition. Combined, these results indicate that determining *CYP3A5* genotypes in pediatric heart transplant recipients represents a key component in individualizing dosing strategies.

Tacrolimus is predominantly (~95%) cleared via CYP3A5 and CYP3A4 in the liver and intestines,^6,7^ suggesting that genetic polymorphisms causing variable protein expression and activity in these enzymes have the potential to dramatically alter tacrolimus PK. Extensive literature showcases the impact of *CYP3A4* and *CYP3A5* genetic variation on tacrolimus PK, including in both adult and pediatric heart transplantation.^17,19,20,22^ Gijsen et al. found a 2.3-fold increase in median tacrolimus dose in pediatric CYP3A5 expressors (i.e. those with the CYP3A5*1 allele) relative to non-expressors,^19^ consistent with the findings of the current study, while a more recent study by Liu et al. identified a 1.5 fold increase.^22^ While the current study could not analyze the impact of the CYP3A4*22 variant, due to the paucity of children in the study with this genotype, Gijsen et al found that children carrying this allele required a 30% lower tacrolimus dose relative to those with the CYP3A4*1/*1 genotype.^20^ In adults, Deinenger et al. observed a 2.1-fold higher dose was required in CYP3A5 expressors, but did not find a significant effect from the CYP3A4*22 allele.^17^ Combined, these data point to a clear rationale for *CYP3A5* and *CYP3A4* genotype-guided tacrolimus dosing to more efficiently attain stable tacrolimus concentrations within the expected therapeutic range.

Despite the existing evidence of the impact of *CYP3A4/5* genetics on tacrolimus PK, there is limited implementation of these findings in clinical practice. Indeed, the most recent guidelines for tacrolimus TDM acknowledge the impact of CYP3A4/5 expression on dose requirements, but stops short of recommending genotype-guided dosing due to limited data on the benefits of this approach for clinical outcomes.^5^ A recent study within our institution incorporated pharmacogenetics testing in conjunction with Clinical Pharmacogenetics Implementation Consortium (CPIC) guidelines into the clinical care of pediatric heart and kidney transplant recipients.^30^ Findings from this study suggest that pharmacogenetic-guided dosing potentially (1) limits the incidence of adverse events in this population, (2) impacts dosing of medications beyond tacrolimus, and (3) refines current tacrolimus dosing guidelines.^30^ Further work is needed to assess clinical outcomes, with regards to both efficacy and toxicity, to determine the value of pharmacogenetic-guided tacrolimus dosing and support its implementation into clinical practice. However, it is anticipated that pharmacogenetic-guided dosing will, at a minimum, decrease the time to stable therapeutic tacrolimus dosing for the child, with tangible benefits in reduced hospitalization stay length, number of venipuncture samples collected for TDM, and overall healthcare system costs.

Beyond demonstrating the impact of *CYP3A5* genotype on tacrolimus dose requirements, the current study identified genotype-associated differences in metabolite concentrations. Both tacrolimus and its M-I metabolite were present at higher concentrations in those with the CYP3A5*1 allele, consistent with the functional protein and higher dose requirement associated with this genotype. This relationship was inverted when normalized by the weight-adjusted totally daily dose, suggesting that the increased metabolism associated with the CYP3A5*1 genotype is likely attenuated by the increased doses administered in these children following clinical TDM results. In a small study of adult kidney transplant recipients, dose-normalized tacrolimus, M-I, and M-II concentrations were lower in CYP3A5 expressors,^16^ in agreement with the current study. In a study of adult kidney transplant recipients, the ratio of tacrolimus M-I to tacrolimus area under the curve was found to be 178% greater in CYP3A5 expressors relative to non-expressors,^23^ similar in direction to the findings in the current study when only baseline data were considered. Importantly, prior work suggests that tacrolimus metabolites may bind to the same receptor as tacrolimus (FKBP12) but do not confer any immunosuppressive activity or cause pharmacodynamic interactions with the parent tacrolimus molecule,^31^ indicating that increased metabolite formation in CYP3A5 expressors is unlikely to impact the efficacy/toxicity associated with tacrolimus use. Interestingly, the current study yielded a hypothesized tacrolimus:M-I concentration ratio of <6.6 to be predictive of possessing a CYP3A5*1 allele. While work is needed to refine and confirm this threshold, this metabolite ratio phenotype may provide an alternative approach to determining *CYP3A5* genotype that can be completed as part of standard clinical TDM analyses.

Population pharmacokinetic modeling further supported the role of *CYP3A5* genotype as a significant covariate, with carriers of the CYP3A5*1 allele exhibiting a markedly higher elimination rate. Incorporating genotype into the existing model is expected to improve the model’s predictive performance, however, there was insufficient overlap between this and prior studies to allow this evaluation. Future studies confirming the predictive impact of *CYP3A5* genotype within our current model are needed and may allow a further reduction in time to stable tacrolimus therapeutic dosing than ~3 days found in prior work with the model.^26^ Further reducing the time to stable therapeutic tacrolimus dosing in the immediate post-transplant period may have positive impacts on clinical outcomes (efficacy or toxicity) and reduce healthcare system burden for transplant recipients and their providers.

Despite the strengths of the current study, a few limitations merit consideration. First, despite enrolling a sufficient number of children with the CYP3A5*1 allele, the study population was predominantly White, which may limit generalizability to more diverse populations where allele frequencies differ. Second, while strong associations between genotype and tacrolimus/metabolite concentrations were observed when considering all collected data, these data are not fully independent, as 3 to 4 samples were collected per child. When reduced to analyzing baseline sample data, the significance of some of the associations were attenuated, likely due to the smaller sample size. Third, the small number of participants carrying the CYP3A4*22 allele precluded an appropriately powered analysis of its impact, and future studies should explore the combined effect of multiple genetic variants on tacrolimus PK. These limitations point to the need for additional larger, multicenter studies to confirm our findings and meet the evidence burden required for clinical implementation of genotyping informed tacrolimus dosing.

In summary, this study provides compelling evidence that *CYP3A5* genotype significantly influences tacrolimus PK and dose requirements in pediatric heart transplant recipients. Incorporating genotype into initial dosing decisions (with or without population PK driven dose guidance) represents a useful strategy to individualize immunosuppressive therapy and may improve clinical outcomes in this important population.

## Figures and Tables

**Table 1:** Study population demographics

Study Period	9/2020 – 8/2023
Subjects	64
Sex	44 Male, 20 Female
Race	59 White, 3 Black, 2 Other
Fluconazole Use	61 No, 3 Yes
*CYP3A4* Genotype	60 *1/*1, 3 *1/22, 1 *22/*22
*CYP3A5* Genotype	2 *1/*1, 14 *1/*3, 48 *3*3
Age (year)	
*Median (IQR)*	9.7 (6.0, 137)
Weight (kg)	
*Median (IQR)*	29.5 (17.5, 46.4)
Creatinine Clearance (mL/min/1.73m ^2^ )	
*Median (IQR)*	105 (93.2, 123)
Time After Transplant (year)	
*Median (IQR)*	5.3 (1.9, 8.1)
Dose (mg/kg/day)	
*Median (IQR)*	0.0789 (0.0515, 0.118)
[Tacrolimus] (ng/mL)	
*Median (IQR)*	6.0 (4.2, 8.5)

IQR, interquartile range

## Data Availability

Data that can be reasonably shared without compromising a participant’s identity can be made available upon request.

## Article Summary

### What is known:

1. Tacrolimus is a primary immunosuppressant agent in preventing graft rejection in children receiving heart transplant.
2. Tacrolimus suffers from substantial inter- and intra-patient pharmacologic variability, complicating dosing strategies, in part due to genotype dependent metabolism.

### What this study adds:

1. The CYP3A5*1 allele is associated with a 2.7-fold increase in tacrolimus dose requirement to maintain therapeutic concentrations.
2. A concentration ratio of tacrolimus to its 13-O-desmethyl (M-I) metabolite may predict genotype.
